# Glycine supplementation extends lifespan of male and female mice

**DOI:** 10.1111/acel.12953

**Published:** 2019-03-27

**Authors:** Richard A. Miller, David E. Harrison, C. Michael Astle, Molly A. Bogue, Joel Brind, Elizabeth Fernandez, Kevin Flurkey, Martin Javors, Warren Ladiges, Christiaan Leeuwenburgh, Francesca Macchiarini, James Nelson, Alexey G. Ryazanov, Jessica Snyder, Timothy M. Stearns, Douglas E. Vaughan, Randy Strong

**Affiliations:** ^1^ Department of Pathology, Paul F. Glenn Center for Biology of Aging Research University of Michigan Ann Arbor Michigan; ^2^ The Jackson Laboratory Bar Harbor Maine; ^3^ Department of Natural Sciences, Baruch College CUNY New York New York; ^4^ Natural Food Science, LLC New Hamburg New York; ^5^ Department of Pharmacology, Barshop Institute for Longevity and Aging Studies, Geriatric Research, Education and Clinical Center and Research Service, South Texas Veterans Health Care System The University of Texas Health Science Center at San Antonio San Antonio Texas; ^6^ Department of Psychiatry University of Texas Health Science Center San Antonio Texas; ^7^ Department of Comparative Medicine, School of Medicine University of Washington Seattle Washington; ^8^ Department of Aging and Geriatric Research, Division of Biology of Aging, Institute on Aging, College of Medicine University of Florida Gainesville Florida; ^9^ Division of Aging Biology National Institute on Aging Bethesda Maryland; ^10^ Department of Cellular and Integrative Physiology, Barshop Center for Longevity and Aging Studies The University of Texas Health Science Center at San Antonio San Antonio Texas; ^11^ Department of Pharmacology Rutgers Robert Wood Johnson Medical School Piscataway New Jersey; ^12^ Princeton Institute of Life Sciences Princeton New Jersey; ^13^ Department of Medicine Northwestern University Feinberg School of Medicine Chicago Illinois

**Keywords:** anti‐aging, life span, longevity regulation

## Abstract

Diets low in methionine extend lifespan of rodents, though through unknown mechanisms. Glycine can mitigate methionine toxicity, and a small prior study has suggested that supplemental glycine could extend lifespan of Fischer 344 rats. We therefore evaluated the effects of an 8% glycine diet on lifespan and pathology of genetically heterogeneous mice in the context of the Interventions Testing Program. Elevated glycine led to a small (4%–6%) but statistically significant lifespan increase, as well as an increase in maximum lifespan, in both males (*p* = 0.002) and females (*p* < 0.001). Pooling across sex, glycine increased lifespan at each of the three independent sites, with significance at *p* = 0.01, 0.053, and 0.03, respectively. Glycine‐supplemented females were lighter than controls, but there was no effect on weight in males. End‐of‐life necropsies suggested that glycine‐treated mice were less likely than controls to die of pulmonary adenocarcinoma (*p* = 0.03). Of the 40 varieties of incidental pathology evaluated in these mice, none were increased to a significant degree by the glycine‐supplemented diet. In parallel analyses of the same cohort, we found no benefits from TM5441 (an inhibitor of PAI‐1, the primary inhibitor of tissue and urokinase plasminogen activators), inulin (a source of soluble fiber), or aspirin at either of two doses. Our glycine results strengthen the idea that modulation of dietary amino acid levels can increase healthy lifespan in mice, and provide a foundation for further investigation of dietary effects on aging and late‐life diseases.

## 1 INTRODUCTION

Experiments to find drugs or nutritional supplements that can extend healthy lifespan in mice have four main goals. First, they provide new models for testing the idea that interventions can retard multiple aspects of age‐dependent decline, including lethal illnesses and changes that impair health but seldom lead to death. Second, they give specific biochemical and physiological clues as to the nature of processes that regulate the pace of age‐dependent decline in multiple systems. Lifespan extension and health preservation in rapamycin‐treated mice, for example, have prompted valuable studies into the role of mTOR, the target of rapamycin, in normal mice and in mice with unusual susceptibility to organ‐specific illnesses (Harrison et al., 2009; Miller et al., 2014; Wilkinson et al., 2012). Third, they help guide the search for additional interventions with potential beneficial effects. Beneficial effects of acarbose, which inhibits postprandial glucose spikes, can, for example, motivate new studies of drugs that also modulate glucose transients. Lastly, data on longevity and health outcomes in mice can serve to guide and inspire parallel searches for interventions that might, hypothetically, postpone multiple aspects of age‐related decline in humans.

The NIA Interventions Testing Program (ITP) has to date reported on four drugs with consistent major effects on mouse lifespan in one or both sexes and found evidence for significant but less dramatic effect of four other drugs. Rapamycin, started at 9 months of age, was found to increase median lifespan by as much as 26% in females and 23% in males, and to retard many aspects of age‐related pathological change (Harrison et al., 2009; Miller et al., 2014). Surprisingly, similarly strong lifespan effects were seen even in mice not given rapamycin until 20 months of age (Harrison et al., 2009). Acarbose can lead to an increase of 22% in median lifespan in male mice, and to a significant, but smaller, 5% increase in female mice (Harrison, 2014; Strong, 2016). Both rapamycin and acarbose improve longevity in the oldest age‐groups, as indicated by a statistical test that compares the proportion of control and drug‐treated mice surviving to the 90th percentile age of the joint distribution, the Wang/Allison test (Wang, Li, Redden, Weindruch, & Allison, 2004). Acarbose produces significant longevity increases, including survival to the 90th percentile, in both sexes, when started as late as 16 months of age (Strong, 2016). A third drug, 17‐α‐estradiol (17aE2), a nonfeminizing congener of the well‐known estrogen 17‐β‐estradiol, increases lifespan of male mice by 19% (Harrison, 2014; Strong, 2016) and has a significant effect on survival to the 90th percentile age, but has no significant effect on female mice. Male mice given 17aE2 live significantly longer than female mice whether or not the females have been exposed to 17aE2. Lastly, NDGA (nordihydroguaiaretic acid) has been shown to increase lifespan of male mice only, with an increase of 12% in median in two independent experimental groups (Strong, 2016; Strong et al., 2008), without a significant effect in female mice. Nordihydroguaiaretic acid, at the doses used, did not lead to significant changes in survival to the 90th percentile age.

Of the other agents tested so far by the ITP, four (methylene blue, aspirin, Protandim, and green tea extract [GTE]) provided some evidence for possible health benefits. Methylene blue (Harrison, 2014) led to a significant (*p* = 0.004) increase in maximum lifespan that was limited to females and was not accompanied by alteration in median. Aspirin produced an 8% increase in median lifespan (*p* = 0.01) that was seen only in male mice, with no significant change in maximal lifespan by the Wang/Allison test (Strong et al., 2008). Protandim, an inducer of the stress‐resistance factor Nrf2, led to a 7% increase in median lifespan in male mice (Strong, 2016), but there was no effect in females, no effect on maximum lifespan in either sex, and a dramatic site‐to‐site variance, with strong lifespan effects seen only at the University of Texas Health Science Center at San Antonio (UT) but not at the Jackson Laboratory (TJL) or University of Michigan (UM) sites. Green tea extract (Strong et al., 2013) did not show a significant effect by our standard analyses in either sex, but may have conferred some benefit, in females, against early and mid‐life deaths (*p* = 0.03 by Wilcoxon–Breslow test, which gives greater weight to early than to later deaths).

Diets low in the amino acid methionine have been shown to extend median and maximum lifespan in rats (Orentreich, 1993; Richie et al., 1994; Zimmerman, 2003) and in mice (Brown‐Borg et al., 2014; Miller et al., 2005; Sun, 2009), although it is not yet clearly established if diets deficient in other single amino acids might also lead to similar benefits. As a practical matter, interventions that involve supplementation of specific nutrients would be easier to test, in humans or mice, than diets that require depletion of specific compounds. Glycine plays a special role in methionine metabolism, serving as the only acceptor for methyl groups, through action of glycine‐N‐methyl transferase (GNMT), the key enzyme in the only pathway for methionine clearance in mammals (Luka, Mudd, & Wagner, 2009). Methionine toxicity can be blocked by dietary glycine (Luka et al., 2009), consistent with the notion that GNMT is the principal effector of methionine clearance, at least at toxic levels. These data suggest that excess dietary glycine might depress methionine levels and thus mimic some of the benefits of a low methionine diet. Glycine‐supplemented diets have been reported to produce anticancer and anti‐inflammatory effects in rodents (Alarcon‐Aguilar, 2008; Wang et al., 2013; Zhong et al., 2003) and to provide benefits in humans with type II diabetes during a 3‐month trial (Cruz, 2008). A small study using glycine supplementation in Fisher 344 rats showed significant lifespan extension at levels of 8%, 12%, and 20% (Brind, 2011), although the higher glycine levels led to weight loss compared to control rats. Maximum lifespan, evaluated by the Wang/Allison method, was significantly increased (*p* = 0.03) only at the 8% supplementation level. In this earlier experiment, glycine treatment did not elevate plasma methionine levels, suggesting that the effect was not due to minimizing methionine toxicity, but to some other, unknown, mechanism.

We have now conducted a lifespan trial of glycine supplementation in a large group of genetically heterogeneous male and female mice, and report here that 8% glycine leads to significant increases in longevity in both sexes and at each of three independent test sites. In the same annual cohort, we found no alteration of lifespan in mice treated with aspirin (60 or 200 ppm), inulin (600 ppm), or TM5441 (an inhibitor of plasminogen activator inhibitor 1; used at 60 ppm.).

## 2 RESULTS

### 2.1 Increased longevity in male and female mice

**Figure 1:**
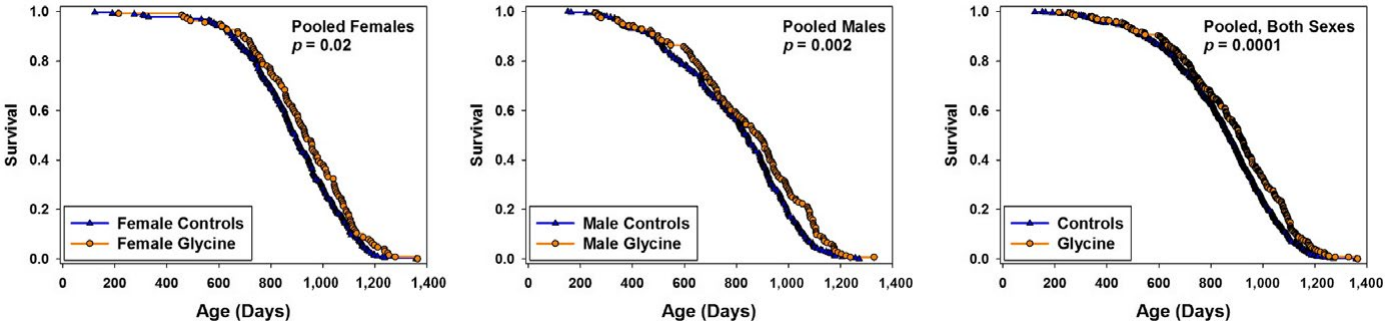
Survival curves for glycine‐treated mice, pooled across sites. Each symbol represents one mouse. *p*‐values calculated by log‐rank test, stratified by site (for panels with single‐sex data) or by both site and sex (for the panel in which both sexes are combined.)

Mice given supplemental glycine as 8% of their diet by weight, from age 9 months, survived significantly longer than controls. Figure 1 shows survival curves separately for males and for females, as well as the survival curve for both sexes pooled together. Our principal statistical criterion, as in earlier ITP studies, is the log‐rank test evaluated separately for each sex (with stratification for site). As shown in Table 1, *p* = 0.006 for females and *p* = 0.002 for male mice by this criterion. When males and females are combined, the log‐rank test gives *p* = 0.00004 with stratification for site and sex. The effect on median lifespan is small: Median survival age increases only 3% in each sex, calculated as the average change across the three sites. When all the data are pooled for each sex, the change in median is 4% in females and 6% in males. The average increase in the age at which 90% of the mice have died is 2% for females and 6% for males, calculated as the mean of this value among the three sites. Our statistical criterion for questions about “maximum lifespan” is the test proposed by Wang and Allison (Wang et al., 2004), which compares the number of mice in each group that remains alive at the age at which 90% of the mice in the pooled life table have died. By this test, *p* = 0.7 for females, *p* = 0.0005 for males, and *p* = 0.006 for the combination of males and females. We conclude that 8% glycine diet causes a small lifespan extension in both sexes, with late‐life effects seen at least in males.

**Table 1:** Survival statistics for glycine effect, pooled, and site‐specific

Sex	Rx	Log‐rank *p*‐value^a^	Median	Change in median^b^	Site average^c^	P90^d^	Change in P90^e^	Site average (P90)^f^	WA *p*‐value^g^	Count
Pooled across sites										
Female	Control		897			1,109				280
Female	Glycine	0.006	929	3.7%	3.2%	1,129	1.8%	2.4%	0.7	136
Male	Control		832			1,059				300
Male	Glycine	0.002	884	6.2%	2.9%	1,107	4.5%	6.2%	0.0005	156
Both	Glycine	0.00004							0.006	
TJL										
Female	Control		904			1,099				96
Female	Glycine	0.01	959	6		1,190	8		0.04	48
Male	Control		873			1,068				102
Male	Glycine	0.3	864	−1		1,107	4		0.27	54
Both	Glycine	0.013							0.02	
UM										
Female	Control		884			1,139				92
Female	Glycine	0.2	925	5		1,129	−1		0.77	44
Male	Control		894			1,084				99
Male	Glycine	0.1	901	1		1,138	5		0.13	51
Both	Glycine	0.053							0.52	
UT										
Female	Control		882			1,109				92
Female	Glycine	0.8	872	−1		1,106	0		1.00	44
Male	Control		743			977				99
Male	Glycine	0.006	810	9		1,073	10		0.04	51
Both	Glycine	0.026							0.83	

a Log‐rank *p*‐values are stratified by site when single‐sex data are evaluated and stratified by site and sex when data from males and females are combined.

b Change in median is calculated as [Median for Glycine minus median for Controls] divided by [Median for controls] pooled across the three test sites, calculated separately for each sex, and expressed as a percentage.

c “Site average” is the mean value of the three site‐specific values for change in median.

d P90 is the age at which 90% of the mice had died.

e “Change in P90” shows percent difference between glycine and control mice, separately for each sex.

f “Site average” for P90 is the mean value from the three site‐specific calculations of P90.

g WA *p*‐value is derived from Fisher's exact test version of the Wang/Allison procedure, a test of distribution of control versus glycine mice at the age at which 90% of mice have died in the joint survival distribution. The number of expected and observed dead is evaluated separately at each site (and for each sex, when pooling over sex), and the counts then added for Fisher's exact test statistic.

The site‐specific data in Table 1 show that glycine caused an increase in lifespan at each site, with *p* = 0.013, *p* = 0.053, and *p* = 0.026 at TJL, UM, and UT, respectively, when male and female data are combined. The effect was stronger for females at TJL and stronger for males at UT in this experiment.

**Figure 2:**
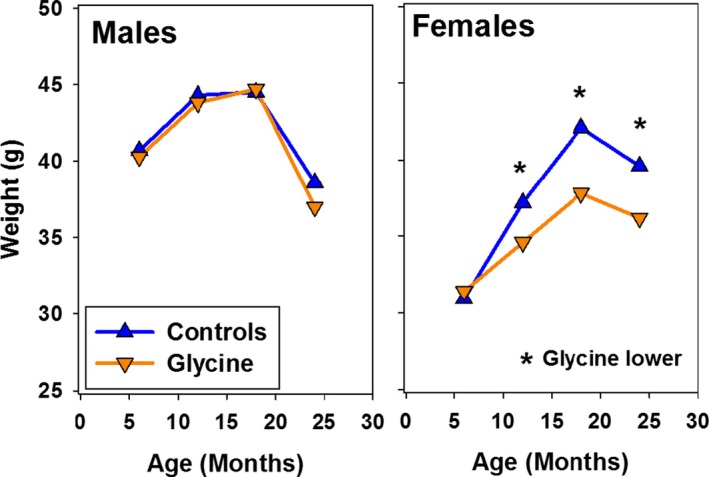
Weights at 6, 12, 18, and 24 months for glycine‐treated mice, pooled across sites. Symbols show mean values. For controls, *N* ~ 96, 91, 83, 70 of each sex at each site, at the ages of 6, 12, 18, and 24 months, respectively, and *N* for each group of glycine mice is about 50% of the number of controls. *SEM* values (not shown) are <1 g for each age/treatment group, except *SEM* = 1.6 g for glycine‐treated male mice at 24 months. Drug effect was evaluated by a two‐factor ANOVA (site, drug, with interaction term). The effect of glycine was *p* > 0.4 for males at each age. For females, *p* < 0.004 at ages 12, 18, and 24 months, as indicated by the asterisks.

The glycine‐supplemented diet led to lower weights in female, but not in male, mice, as shown in Figure 2. By 12 months of age, that is, 3 months after initial exposure to the high glycine diet, female mice were significantly lighter in weight than control animals (2.6 g, equivalent to 7% of control weight). Glycine‐treated female mice were 10% lighter than controls at 18 months, and 9% lighter at 24 months, in each case significantly lower than in control females. Although it is possible that diminished food intake, and thus some form of caloric restriction, may have affected health in glycine‐treated females, this explanation seems unlikely to apply to male mice, in which lifespan effects were equivalent and in which the diet did not alter weight trajectory.

### 2.2 End‐of‐life pathology

Mice in the glycine and control groups were selected (by random numbers) for necropsy analysis. Tissue sections were evaluated by a veterinary pathologist (JMS) who was unaware of group assignment. “Cause of death” diagnoses are summarized in Table 2. Of the 119 specimens submitted, 8 were too severely autolyzed to be evaluated, and another 11 showed advanced autolysis but with evidence of lethal neoplasm (details in Note d of Table 2). Of these 19 autolysis cases, 15 came from a single site (TJL), so TJL cases are somewhat under‐represented in the remainder of the table. Of the 100 nonautolyzed cases (53 controls and 47 glycine‐treated), a presumptive cause of death could be inferred in 81; the other 19 included 4 in which multiple potentially fatal diseases were present (see Note c in Table 2). and 15 in which no lesion or set of lesions was judged to be clearly responsible for the death of the animal.

**Table 2:** Summary of cause of death diagnoses

Cause of death	Controls	Glycine	*p*‐value
Hemangiosarcoma	2	1	
Hepatocellular carcinoma	4	3	
Lung adenocarcinoma	9	2	0.03
Mammary adenocarcinoma	1	2	
Metastatic carcinoma	1	5	0.098
Neoplasm (other)^a^	2	2	
Hematopoietic neoplasia (Round cell tumor, RCT)^b^	12	11	
Sarcoma (other)	2	2	
Soft tissue sarcoma	3	5	
Amyloidosis	1	0	
Atrial thrombosis	2	3	
Endometritis/metritis	2	0	
Hepatic necrosis	0	1	
Nephritis	2	1	
Multiple processes^c^	3	1	
Open	7	8	
Total (not autolysis)	53	47	
Autolysis	4	4	
Autolysis (likely neoplasm)^d^	2	9	0.03

a Harderian gland adenocarcinoma; a necrotic, hemorrhagic, and thrombosed large subcutaneous neoplasm; a multifocal thoracic neoplasm with characteristics of both sarcoma and carcinoma; and a liver liposarcoma.

b 17 probably lymphomas; 6 probably histiocytic sarcomas; immunohistochemistry not performed.

c 1 severe amyloidosis and lung adenocarcinoma; 1 pituitary adenoma and atrial thrombosis; 1 lung adenocarcinoma and stomach squamous cell carcinoma; and 1 RCT and pituitary adenoma.

d 7 likely to be hematopoietic (RCT); 1 hepatocellular carcinoma, 1 lung carcinoma, 1 sarcoma, and 1 hemangiosarcoma.

In the 81 cases in which a likely cause of death was assigned, glycine‐treated mice were significantly less likely than controls to die of pulmonary adenocarcinoma limited to the lung (*p* = 0.03). This is a lesion that is more often seen in males (seven cases) than in females (four cases). In addition, we noted that adenocarcinoma limited to the lung, as a cause of death, was seen frequently at TJL (five cases) and at UT (five cases) but rarely at UM (one case); we do not understand the reason for this site‐specific effect. Cases with lethal metastatic carcinoma, with an unknown primary source, were somewhat more common in the glycine group, but the difference did not reach statistical significance (*p* = 0.098). Some of these may have had lung or liver as a primary source, which might be evaluated by additional immunohistochemical methods.

In addition to evaluation of cause of death, the pathologist noted “incidental” lesions that were present at time of death but were not thought to be sufficiently severe to have led to death or terminal morbidity. Supporting Information Table S2 lists 12 lesions that were evaluated on a graded scale, and Supporting Information Table S3 lists the 28 lesions that were listed as present or absent. There were no significant differences between control and glycine‐treated mice for any of these conditions.

### 2.3 Aspirin, inulin, and TM5441 did not lead to lifespan effects

**Figure 3:**
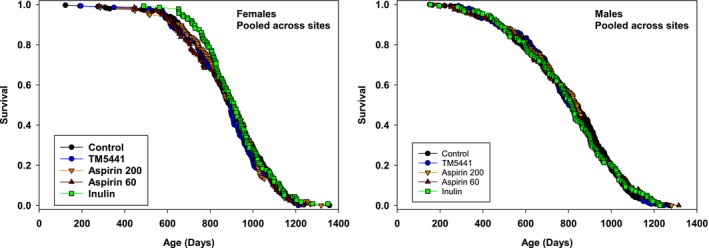
Survival curves for mice treated with aspirin, inulin, or TM5441, pooled across sites. Left panel: females, pooled across sites. Right panel: males, pooled across sites

Many interventions proposed and accepted by the ITP for longevity testing do not lead to increased lifespan, and ITP policy is to report these negative effects for all compounds tested. Using the same control mice that were evaluated in the glycine experiment, we evaluated the possible effects of aspirin (at doses of 60 and 200 ppm), inulin (at 600 ppm), and TM5441, an inhibitor of PAI‐1, used at 60 ppm. Each of these was given to the mice starting at age 11 months. Mice were tested at all three sites in roughly equal numbers, as in the glycine protocol. As shown in Figure 3 and Supporting Information Table S4, none of these treatments led to a significant alteration in longevity in male or female mice, either by our principal criterion the log‐rank test, or when evaluated by the Wang/Allison method at the 90th percentile. None of these agents had any significant effect on weight of male mice (not shown). Weight of female mice was unaffected by aspirin or inulin, but female mice treated by TM5441 were significantly heavier than control females at ages 12, 18, and 24 months (*p* < 0.02 at each age; data not shown).

### 2.4 Assessment of drug and glycine levels in treated mice

Four batches of food containing supplemental glycine were evaluated for glycine content, with an average value of 7.1%, which is 88% of the nominal expected level of 8% glycine. In a pilot experiment, UM‐HET3 mice were given a glycine‐supplemented diet from 10 months of age for a period of 12 weeks. Serum levels of glycine in these glycine‐treated mice were 54 ± 18 μg/ml (mean ± *SD*; *N* = 4), and thus sixfold higher than glycine levels of control mice, which were 9 ± 0.9 μg/ml (*N* = 4). Similarly, liver levels of glycine were 1,200 ± 700 ng/mg in the treated mice, fourfold higher than in controls (290 ± 40 ng/mg). A second experiment using a separate set of mice produced similar results: 85 versus 14 μg/ml in serum and 1,900 versus 335 ng/mg in liver. The correlation between serum and liver glycine values in the glycine‐fed mice had *R* ^2^ = 0.94; mice with high serum glycine levels were also relatively high in their liver glycine content. Thus, glycine supplementation of food led to an increase in this amino acid in both serum and liver tissue.

Aspirin and its breakdown product salicylic acid (SA) were measured in three independent batches of food pellets, and the sum of [aspirin plus SA] was found to be 83% of expected in the pellets nominally containing 60 ppm, and 93% of expected levels in pellets nominally containing 200 ppm. In a pilot experiment, UM‐HET3 mice were given chow containing aspirin for 11 weeks, starting at 6 months of age. Aspirin is quickly converted to SA once absorbed in the small intestine, and mean serum levels of SA in the mice given 60 ppm were 1.21 ± 1.03 μg/ml (mean ± *SD* for *N* = 17), and were 4.1 ± 4.4 in mice given 200 ppm (*N* = 21). SA levels in mice fed control chow were consistently less than the detection limit of 0.15 μg/ml.

Three batches of food were tested for levels of TM5441, and the mean level of drug was 85% of the nominal value. Serum levels of TM5441 were 198 ng/ml (*N* = 7, *SD* = 71) when mice were given chow containing TM5441 for 11 weeks starting at six months of age. As expected, serum from control mice did not contain detectable levels of TM5441 and is thus below the standard curve limit of 1.6 ng/ml.

## 3 DISCUSSION

Our most interesting new finding is that glycine‐supplemented diets extend lifespan of male and female UM‐HET3 mice, including significant benefits in lifespan to mice at the 90th percentile age. This supplementation leads to significant effects at each of the three test sites. The result is consistent with an earlier report (Brind, 2011) showing a beneficial effect of glycine on lifespan (including maximal lifespan) in small groups of Fisher 344 rats. The effect in mice, although consistent with this prior rat study, is quite small and does not much alter median survival age. We speculate that variations in this protocol, for example, in which glycine supplementation is started earlier or terminated once mice are fully grown, might have improved effects on health and lifespan.

Pathology assessment showed that UM‐HET3 mice at end of life have a wide variety of age‐related lesions similar to those identified in previous longitudinal studies in mice (Harrison, 2014; Snyder, Ward, & Treuting, 2016). Incidental degenerative lesions, presumably related to aging but not a primary cause of death, occurred in all major organs examined, but did not appear to be influenced by dietary glycine supplementation. Thus, there is no evidence from this work that glycine supplementation impairs health. Autolysis of organs secondary to carcass preservation technique and postmortem autolysis in animals found dead complicated the scoring of degenerative lesions in many organs. A cross‐sectional study, in which mice within a treatment cohort are euthanized and promptly subjected to necropsy with organ removal can be compared with a control cohort at the same time point, might show treatment differences, though such a design provides little information about cause of death and tends to underestimate the incidence of lesions that lead to death quickly (Wolf, Giddens, & Martin, 1988).

Of the 81 cases in which a presumptive cause of death was determined, 71 (88%) died as the result of neoplasia, which is similar to previous studies (Harrison, 2014; Lipman, 2004; Miller & Chrisp, 2002). Dietary glycine had no effect on these causes of death, except for pulmonary adenocarcinoma, where only two mice died in the glycine‐supplemented cohort, compared to nine dying from this cancer type in mice fed the control diet (*p* = 0.03). If one includes the two mice (one in each group) in which pulmonary adenocarcinoma was considered to be a contributing cause of death, the difference in proportion reaches *p* = 0.06. Pulmonary adenomas were also seen more frequently in control animals (a total of 12 neoplasms in nine mice) compared to glycine‐treated animals (a total of seven neoplasms in seven mice), although the difference between groups was not statistically significant. Several mice, especially in the glycine group, had metastatic carcinomas which affected the lung in addition to a variety of other organs. Immunohistochemistry and other testing to better characterize the tissue of origin of these neoplasms were not performed, but one or more of these neoplasms may have arisen from the lung. There was no significant difference between the control and glycine groups when all carcinomas involving the lung, including metastatic neoplasms, were considered. Cross‐sectional studies in which neoplasms are potentially identified at an earlier stage may help define whether glycine exerts a protective effect on the development of pulmonary carcinoma. Hematopoietic neoplasms (round cell tumors) were also a common cause of death in mice in this study, a category that includes lymphomas and histiocytic sarcomas. Immunohistochemistry was not performed to definitively distinguish between the two. No glycine effect was seen on the frequency of hematopoietic tumors in these end‐of‐life necropsies.

Glycine is considered a nonessential amino acid, because it can be synthesized by several pathways, and hence dietary intake provides only part of the glycine pool. The endogenous glycine pool is also affected by glycine clearance pathways. A preliminary life‐span study in rats using glycine supplementation (Brind, 2011) was originally conceived as an approach to mimic the well‐established life‐extending effect of methionine restriction (Brown‐Borg et al., 2014; Miller et al., 2005; Orentreich, 1993; Richie et al., 2004; Sun, 2009). The strategy was based on the understanding that glycine is the only acceptor for methyl groups by the action of glycine‐N‐methyl transferase (GNMT), and that in turn GNMT represents the only pathway for the clearance of excess methionine in mammals (Luka et al., 2009). Supplemental glycine, however, was found (Brind, 2011) not to reduce plasma methionine levels, in contrast to the 60% reduction in plasma methionine levels seen in rats on a low methionine diet. This result suggested that elevated glycine levels, in plasma or tissues, might in themselves produce health benefits and increased longevity. Glycine receptors are gated chloride channels that hyperpolarize plasma membranes and can inhibit cell activation; although they were originally thought to be restricted to the nervous system, more recent work has demonstrated glycine receptors on many cell types, including macrophages of lung and liver, and platelets (Schemmer et al., 2013; Wheeler, 1999). Glycine promotes chloride influx largely at concentrations (0.5–1 mM) that are considerably higher than those needed for protein synthesis and one‐carbon transfer reactions. Prevention of cell activation and depolarization by maintenance of resting membrane potentials may help to limit macrophage activation (Zhong et al., 2003).

Anti‐inflammatory, anticancer, and other putative benefits of glycine supplementation have been demonstrated in rodent models (Alarcon‐Aguilar, 2008; Wang et al., 2013; Yi, Xu, & Allison, 2003), and one translational study noted benefits in human diabetes trials (Cruz, 2008). A small pilot study had documented a significant lifespan benefit in inbred rats (Brind, 2011), but our current study provides more definitive evidence by using a second species, by using genetically heterogeneous animals, by documenting significant effects in both males and females, and by showing similar benefits at each of three independent test sites. The mechanism of effect is not yet known and will require additional targeted work, but could conceivably involve lowered inflammation in one or more tissue beds, or effects of glycine on CNS processes, or effects, direct or indirect, on multiple forms of lethal neoplastic disease. Consistent with these ideas, observational studies have documented lower glycine levels in people suffering from diabetes and prediabetes (Guasch‐Ferré et al., 2016), cardiovascular disease (Ding, 2015), and hepatocellular carcinoma (Osman, 2017). Similarly, a recent metabolomics study (Hartiala, 2016) reported a 12% decreased risk of coronary artery disease (CAD) among female human subjects associated with the genetic variant Rs715, which impairs function of the mitochondrial enzyme carbamoyl phosphate synthase (CPS). Carbamoyl phosphate synthase catalyzes the first step of the urea cycle, removing ammonia derived largely from glycine cleavage. Women with the Rs715 variant have elevated plasma glycine levels, which might potentially contribute to their lower risk of coronary disease.

The present study provides support for the hypothesis that consumption of glycine in the normal diet may be suboptimal for longevity, and that higher levels might be beneficial, perhaps through moderation of inflammatory processes. The limited data from the rat study (Brind, 2011) suggest that higher glycine doses might not produce higher benefits, but it is possible that initiation of glycine supplementation at an earlier age might be worth testing.

We considered the possibility that extended longevity in the glycine‐treated mice might reflect low food intake. This seems unlikely, because male mice show extended longevity without small body size. Food intake is very difficult to measure accurately, because of complications from food wastage into cage bedding, caloric loss in feces, and variability among mice in the same cage. In addition, small mice eat less food than large mice, because they have diminished the need for thermogenesis; thus, an association between size and food intake does not demonstrate a causal connection in either direction. We cannot, however, rule out the possibility that glycine supplementation might have led to low food intake and to extended longevity in female mice. Incorporation of glycine into the diet lowers its calorie density by <1%, which is unlikely to lead to any alteration in nutritional status or health.

The ITP is committed to reporting the outcome of all longevity studies, including those of interventions that did not appear to produce health benefits. Three such agents were tested in the same cohort (C2014) that included the glycine‐supplemented mice.

TM5441 was selected for testing because it is an orally active, small‐molecule inhibitor of PAI‐1, the primary inhibitor of tissue and urokinase plasminogen activators, and thus a primary effector of fibrinolysis and extracellular proteolysis (Boe, 2013). PAI‐1 levels increase with age in mice and humans (Khan, 2017; Testa et al., 2009; Yamamoto et al., 2002) and are increased by obesity, insulin resistance, and inflammation (Alessi & Juhan‐Vague, 2006; Khan, 2017). PAI‐1 is present in atherosclerotic plaques and accumulates with age in murine heart muscle (Sobel, Lee, Pratley, & Schneider, 2006). PAI‐1 appears to contribute to common cardiovascular manifestations of aging. PAI‐1 excess is prothrombotic, and PAI‐1 appears to be an important contributor to arteriosclerosis and vascular stiffness (Lieb et al., 2009). A recently published Mendelian analysis concluded that PAI‐1 plays a causal role in coronary heart disease (Song, 2017). PAI‐1 is elevated in the klotho (kl/kl) mutant mouse and in the BubR1^H/H^ mutant, mice which some have interpreted as models of accelerated aging, and administration of TM5441 extends median lifespan of klotho mice (Eren et al., 2014). We hypothesized that inhibition of PAI‐1 by TM5441 would lead to extended lifespan in normal mice, but found no evidence for higher lifespan in UM‐HET3 mice treated from 11 months of age.

Inulin was selected for testing because it had shown preliminary evidence for lifespan benefit in a large‐scale screen using female B6C3F1 mice. In this unpublished screen, over 1,000 drugs and related molecules were tested in parallel using a shared control group. Drugs were administered from 5 months of age at doses 50%–100% of the maximum recommended therapeutic dose for human patients. In this survey, inulin at 600 ppm was found to extend mean and maximal lifespan by 16%, with *p* = 0.06 for the mean value compared to control females. Inulin is a soluble fiber, derived from chicory root, that is metabolized by gut bacteria, and is hypothesized to promote host health by improvements in the balance of species within the gut microbial populations. There is some previous evidence that inulin can extend rat lifespan (Rozan et al., 2008). Our own data did not provide any evidence for health benefits of inulin when given from 11 months of age to male or female UM‐HET3 mice. It is possible that a beneficial effect of TM5441 or inulin might have been seen had we use a different dose, started at an earlier time, started at a later time, or discontinued exposure to the drug after a period of treatment.

Aspirin was selected for testing as a follow‐up to our previous report (Strong et al., 2008) of lifespan extension in UM‐HET3 male mice given aspirin at 20 ppm from 4 months of age. In the original report, we found no effect of aspirin on female longevity, and the effect on male longevity (8% increase in median, *p* = 0.01 by log‐rank test) represented benefits seen at the two sites (UT and TJL) in which male controls were significantly shorter lived than at the third site (UM). To see whether we could obtain larger and more consistent benefits, we tested mice at doses that were threefold and 10‐fold higher than the dose used in the original ITP report. We found no evidence for the benefit of either dose in male or female animals. While it is possible that we might have seen a benefit for 60 ppm or 200 ppm if we had started at an earlier age (4 months, as in the original report), and possible that authentic beneficial effects at 20 ppm are lost at the higher doses, our preferred interpretation is that the original report reflected a type I error and that aspirin does not reliably confer increased lifespan in these genetically heterogeneous mice.

## 4 METHODS

### 4.1 Mouse husbandry

The ITP protocol for longevity studies has been described extensively elsewhere (Miller et al., 2014; Strong, 2016), with details on source of food, bedding, cage changes, light/dark cycle, and other husbandry details. In brief, mice are bred as the progeny of (BALB/cByJ × C57BL/6J)F1 mothers and (C3H/HeJ × DBA/2J)F1 fathers, so that each mouse is genetically unique and a full sib to all other mice with respect to segregating nuclear alleles. Mice are housed at three males or four females per cage from weaning, without replacement as mice die at older ages. They are given free access to food and water. Purina 5LG6 is used as the base diet from weaning, with drugs and supplements given at the concentrations shown in Supporting Information Table S1. Mice are weighed at ages 6, 12, 18, and 24 months, but are otherwise undisturbed. If fight wounds require that mice be removed for humane reasons, all the mice in the affected cage are removed from the study, so as not to enrich for dominant or nondominant individuals. Mice that are removed from the study, either for fighting or for other technical reasons (e.g., chip ID dysfunction, escape, accidental injury), are treated as known to be alive on the day of removal and lost to follow‐up at that point. Mice are inspected daily, and those which are deemed to be unlikely to live for more than another 24 hr, based on a symptom checklist, are euthanized for humane reasons, with the day of euthanasia taken as the best estimate of the date of natural death for statistical purposes. Date of death is also recorded for those found dead. Sentinel mice are tested for antibodies against pathogenic viruses and for parasites, either annually or quarterly depending on the pathogen, and all such tests were negative at each test site throughout the period of this study.

### 4.2 Estimation of glycine, TM‐5441, aspirin, and salicylic acid in food and tissue samples

Glycine levels were measured in food, serum, and liver tissue using an LC/MS/MS system (a Shimadzu SIL 20A HT autosampler, LC‐20AD pumps, and an AB Sciex API 3200 tandem mass spectrometer with turbo ion spray). Serum standard curves were obtained by spiking normal serum at concentrations from 5 to 160 μg/ml. Liver glycine concentrations were estimated in a similar way using sonicated tissues, with calibration standards between 63 and 1,000 ng/mg. LC/MS/MS methods for quantitating TM‐5441 in serum and in food pellets used standards purchased from Tocris Bioscience (Minneapolis, MN). Estimation of aspirin and salicylic acid levels used standards purchased from Sigma Chemical Company (St. Louis, MO). We were unable to develop an assay for inulin in serum, because inulin consists of a complex mixture of polysaccharides of different molecular sizes.

### 4.3 Terminal necropsies

Mice dying (or euthanized when terminally ill) were fixed using a process in which incisions were made in cranium, thorax, and abdomen, and the carcass immersed in 10% neutral‐buffered formalin, and then kept at room temperature for a period of up to 4 years. Glycine‐treated and control cases were then selected by a random number table, selecting 10 male and 10 female glycine‐treated mice from each test site, plus an equal number of control mice. Each animal was given a code so that the assessment of lesions could be conducted by a veterinary pathologist blinded to the treatment group, and the specimens were then shipped to the Geropathology Research Program at University of Washington for necropsy and histopathological analysis. A detailed description of procedures and diagnostic criteria is given in the Supplemental Methods section.

### 4.4 Statistical testing

Drug effects on lifespan are evaluated by the log‐rank test, stratifying by site, separately for males and females. For analyses combining male and female survival data, the log‐rank test includes stratification by both sex and site. These data sets include mice that have died, mice removed for fighting or other cause, and mice still alive at the time of evaluation. Estimates of median survival age and of the age at which 90% have died include only live and dead, but not removed, mice. Hypotheses about survival to very old age are done by the method of Wang and Allison (Wang et al., 2004), using Fisher's exact test. Effects of interventions on body weight are evaluated separately in each sex by a two‐factor ANOVA (drug, site, and interaction terms), with the *p*‐value for the drug effect taken as the key outcome. For the significance tests of group differences in the proportion of mice with specific forms of pathology, we used the “prtest” (proportions test) module of Stata, which computes a normally distributed *z*‐statistic as the difference between the two proportions divided by the standard error of the difference under the null hypothesis. For significance tests of graded pathology findings, we used the Student's *t* test. Significance was assigned with respect to the arbitrary benchmark of *p* = 0.05 in two‐sided tests, without adjustment for multiple comparisons.

## CONFLICT OF INTEREST

Joel Brind: Natural Food Science, LLC, makes and sells a glycine supplement product.
