# Supplementary material for: Glycine supplementation extends lifespan of male and female mice
Source: Aging Cell. 2019 Mar 27;18(3):e12953. doi: 10.1111/acel.12953 (PMC6516426; doi:10.1111/acel.12953)
Supplement: Supplementary file 1 [file ACEL-18-e12953-s001.docx]

**Supplemental Table 1: doses of test agents used**

| **Agent** | **Age started** | **Dose ppm** | **Dose mg per kg body weight/day** | **Dose mg/mouse/day** |
| --- | --- | --- | --- | --- |
| Aspirin 60 | 11 | 60 | 10.0 | 0.3 |
| Aspirin 200 | 11 | 200 | 33.3 | 1.0 |
| Glycine | 9 | 80000 | 13333.3 | 400.4 |
| Inulin | 11 | 600 | 100.0 | 3.0 |
| TM5441 | 11 | 60 | 10.0 | 0.3 |

**Supplemental Table 2: Incidental Pathology – Graded Lesions**

| **Organ** | **Finding** | **N (Control, Glycine)** | **Control** | **Glycine** | **p(t)** |
| --- | --- | --- | --- | --- | --- |
| Adrenal | Ceroid pigment | 31, 25 | 1.6 | 1.8 | 0.3 |
| Heart | Cardiomyopathy | 48, 52 | 1.5 | 1.3 | 0.3 |
| Kidney | Glomerulonephropathy | 41, 36 | 1.7 | 1.6 | 0.7 |
| Liver | Macrovesicular lipidosis | 40, 38 | 0.2 | 0.2 | 0.6 |
| Liver | Microvesicular lipidosis | 40, 38 | 0.7 | 0.5 | 0.4 |
| Liver | Degeneration/necrosis | 41, 43 | 0.7 | 0.9 | 0.3 |
| Liver | Hyperplasia | 39, 38 | 0.3 | 0.5 | 0.4 |
| Ovary | Atrophy | 16, 15 | 3.4 | 3.3 | 0.6 |
| Pancreas | Atrophy | 23, 20 | 0.5 | 0.7 | 0.6 |
| Salivary | Lymphoid aggregates | 51, 48 | 0.7 | 0.8 | 0.4 |
| Testes | Atrophy/degeneration | 23, 23 | 1.7 | 1.9 | 0.4 |
| Uterus | Cystic endometrial hyperplasia | 23, 16 | 1.9 | 2.1 | 0.4 |

**Supplemental Table 3: Incidental Pathology, Categorical Lesions**

| **Organ** | **Lesion** | **Cases** | **Control Cases** | **Glycine Cases** | **Control**  **(%)** | **Glycine (%)** |
| --- | --- | --- | --- | --- | --- | --- |
| Adipose | Mineralization of mesenteric adipose tissue mass | 13 | 7 | 6 | 12 | 10 |
| Adrenal | Neoplasm | 13 | 7 | 6 | 12 | 10 |
| Adrenal | Spindle cell hyperplasia (subcapsular) | 52 | 28 | 24 | 47 | 40 |
| Atrium | Thrombus | 7 | 3 | 4 | 5 | 7 |
| Heart | Neoplasm | 5 | 1 | 4 | 2 | 7 |
| Kidney | Pyelonephritis/pyelitis | 6 | 4 | 2 | 7 | 3 |
| Kidney | Neoplasm (other) | 11 | 5 | 6 | 8 | 10 |
| Liver | Adenoma | 9 | 3 | 6 | 5 | 10 |
| Liver | Hepatocellular adenoma | 14 | 5 | 9 | 8 | 15 |
| Liver | Hemangiosarcoma | 5 | 4 | 1 | 7 | 2 |
| Liver | Hematopoietic neoplasm | 16 | 9 | 7 | 15 | 12 |
| Liver | Neoplasm (other) | 7 | 3 | 4 | 5 | 7 |
| Liver | Telangectasia/angiectasis | 17 | 7 | 10 | 12 | 17 |
| Lung | Pulmonary adenoma | 16 | 9 | 7 | 15 | 12 |
| Lung | Adenocarcinoma | 23 | 13 | 10 | 22 | 17 |
| Lung | Eosinophilic crystalline pneumonia (severe) | 11 | 6 | 5 | 10 | 8 |
| Mammary | Adenocarcinoma | 4 | 1 | 3 | 2 | 5 |
| Ovary | Neoplasm | 5 | 4 | 1 | 7 | 2 |
| Pancreas | Islet hypertrophy | 10 | 6 | 4 | 10 | 7 |
| Spleen | Neoplasm | 17 | 9 | 8 | 15 | 13 |
| Spleen | Extramedullary hematopoiesis | 28 | 13 | 15 | 22 | 25 |
| Testes | Seminal vesicle adenitis | 6 | 2 | 4 | 3 | 7 |
| Thorax | Hematopoietic neoplasm | 15 | 6 | 9 | 10 | 15 |
| Thyroid | Cold follicles | 74 | 41 | 33 | 69 | 55 |
| Thyroid | Follicular cell hyperplasia | 13 | 5 | 8 | 8 | 13 |
| Various | Soft tissue sarcoma | 11 | 4 | 7 | 7 | 12 |
| Various | Hemangiosarcoma | 8 | 6 | 2 | 10 | 3 |
| Various | Hematopoietic neoplasm in female reproductive tract | 13 | 6 | 7 | 10 | 12 |

**Supplemental Table 4: Survival statistics for aspirin, inulin, and TM5441, pooled across test sites.**

| Sex | Rx | Log-rank  p-value | Median (Days) | Change in Median (%) | Site Average | P90 (Days) | Change in P90 (%) | Site Average (P90) | WA p-value |
| --- | --- | --- | --- | --- | --- | --- | --- | --- | --- |
| Female | Control |  | 897 |  |  | 1109 |  |  |  |
| Female | Asp_60 | 0.7 | 908 | 1 | 0 | 1095 | -1 | -1 | 0.6 |
| Female | Asp_200 | 0.8 | 906 | 1 | 1 | 1124 | 1 | -2 | 0.7 |
| Female | Inulin | 0.4 | 910 | 1 | 2 | 1116 | 1 | -1 | 0.7 |
| Female | TM5441 | 0.3 | 894 | 0 | 0 | 1103 | -1 | -1 | 0.9 |
| Male | Control |  | 832 |  |  | 1059 |  |  |  |
| Male | Asp_60 | 0.6 | 820 | -1 | -4 | 1090 | 3 | 2 | 0.14 |
| Male | Asp_200 | 0.4 | 837 | 1 | 0 | 1076 | 2 | 3 | 0.5 |
| Male | Inulin | 0.7 | 815 | -2 | -1 | 1063 | 0 | 5 | 0.7 |
| Male | TM5441 | 0.7 | 802 | -4 | -3 | 1079 | 2 | 3 | 0.3 |

Log-rank p-values are stratified by site when single-sex data are evaluated, and stratified by site and sex when data from males and females are combined. Number of mice: male controls 99 – 102 per site; female controls 92 – 96 per site; treated males 51 – 54 per site; treated females 44 – 48 per site.
